# How Cardiac Embryology Translates into Clinical Arrhythmias

**DOI:** 10.3390/jcdd8060070

**Published:** 2021-06-13

**Authors:** Mathilde R. Rivaud, Michiel Blok, Monique R. M. Jongbloed, Bastiaan J. Boukens

**Affiliations:** 1 Department of Experimental Cardiology, Amsterdam UMC, University of Amsterdam, Amsterdam Cardiovascular Sciences, Meibergdreef 15, 1105 AZ Amsterdam, The Netherlands; m.rivaud@amsterdamumc.nl; 2 Department of Anatomy & Embryology, Leiden University Medical Center, Einthovenweg 20, 2300 RC Leiden, The Netherlands; m.blok@lumc.nl (M.B.); m.r.m.jongbloed@lumc.nl (M.R.M.J.); 3 Department of Cardiology, Leiden University Medical Center, Albinusdreef 2, 2333 ZA Leiden, The Netherlands; 4 Department of Medical Biology, Amsterdam UMC, University of Amsterdam, Amsterdam Cardiovascular Sciences, Meibergdreef 15, 1105 AZ Amsterdam, The Netherlands

**Keywords:** cardiac development, arrhythmias, re-entry, transcription factors

## Abstract

The electrophysiological signatures of the myocardium in cardiac structures, such as the atrioventricular node, pulmonary veins or the right ventricular outflow tract, are established during development by the spatial and temporal expression of transcription factors that guide expression of specific ion channels. Genome-wide association studies have shown that small variations in genetic regions are key to the expression of these transcription factors and thereby modulate the electrical function of the heart. Moreover, mutations in these factors are found in arrhythmogenic pathologies such as congenital atrioventricular block, as well as in specific forms of atrial fibrillation and ventricular tachycardia. In this review, we discuss the developmental origin of distinct electrophysiological structures in the heart and their involvement in cardiac arrhythmias.

## 1. Introduction

Components of the heart with distinct electrophysiological signatures ensure controlled electrical impulse formation and propagation, and coordinated ventricular activation generating sufficient cardiac output required to maintain body homeostasis. During cardiac arrhythmias, cardiac output decreases, which may cause syncope or sudden cardiac death in case of fast heart rate. The origin of a significant part of these cardiac arrhythmias can be traced to congenital or acquired changes in behavior of the electrophysiological components in the heart. The combination of morphology and ion channel make-up can provide different regions of the myocardium, such as the atrioventricular node, the myocardial sleeve of the pulmonary veins or the right ventricular outflow tract (RVOT), with electrophysiological signatures that distinguish them from the working myocardium. During development, cardiogenic transcription factors guide the processes that shape these components and determine the ion channels expressed within [1,2]. Variations in regulatory genetic regions or mutations in genes encoding these transcription factors may alter their expression levels and predispose them to or even cause cardiac arrhythmias originating in these areas [3,4,5,6,7]. Here, we discuss the role of these transcription factors in atrioventricular node function, arrhythmias based on the presence of accessory pathways and arrhythmias originating in the RVOT or pulmonary veins.

## 2. General Cardiac Development

**Figure 1:**
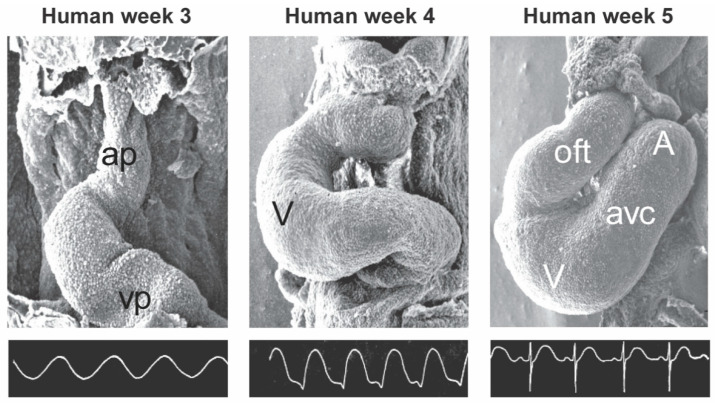
The electrocardiogram of the developing heart. The photographs are scanning electron microscope images of the developing chicken heart of the following stages: from left to right, Hamburger/Hamilton state 11, 14 and 18 correspond to 3, 4 and 5 weeks of human development, respectively. The electrocardiograms are recorded from different chickens of similar developmental stages to the corresponding photographs. These figures are courtesy of S. Virágh and G. Steding. Ap, arterial pole; vp, venous pole; V, ventricle; oft, outflow tract; avc, atrioventricular canal; A, atrium.

The heart forms from a pool of cardiac precursor cells located in a crescent-shaped field of splanchnic mesoderm in the developing embryo. This heart field can be divided into progenitor cells that are located lateral in the embryo and differentiate early into myocardium (referred to as the first heart field) [8], and cells that are located medially and caudally and differentiate later during cardiac development (referred to as the second heart field). The mesenchyme of the first heart field fuses at the midline forming a tube. This early heart tube is composed of primary (embryonic) myocardium and has a venous pole, an atrioventricular canal, a left ventricle and an arterial pole interconnected at the dorsal side by the dorsal mesocardium. At this stage, a large part of the inflow tract, outflow tract and cardiac chambers still need to develop. The cardiomyocytes of the heart tube have a primary (immature) phenotype characterized by an underdeveloped sarcoplasmatic reticulum, weaker contraction and slower conduction compared to cardiomyocytes of the adult heart [9,10]. The slow conduction in the primary myocardium gives rise to a sinusoidal electrocardiogram (Figure 1) [11] and a peristaltic contraction pattern.

**Figure 2:**
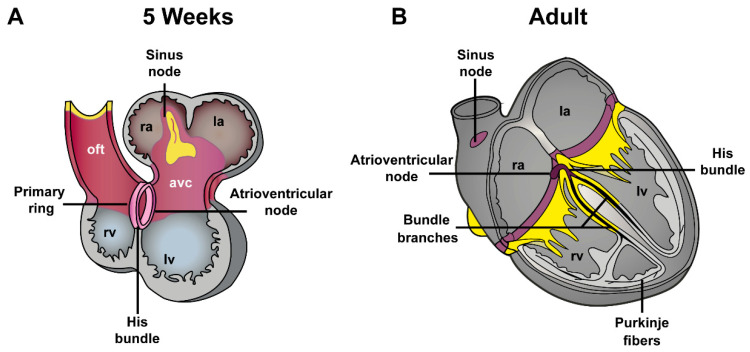
Phenotypes of the embryonic and adult heart (**A**). The embryonic myocytes in the early heart tube possess a phenotype typical for the conduction system with a high automaticity and a low conduction velocity, contractility, and sarcoplasmic reticulum activity. (**B**). The chambers balloon out from the initial heart tube and immediately initiate a fast conducting working myocardial phenotype. The myocardium at the venous pole, and the region interposed between the developing chambers, the atrioventricular canal, initially retains the conduction system phenotype and will form the cardiac conduction system. ra, right atria; la, left atria; oft, outflow tract; rv, right ventricle; lv, left ventricle.

Further in development, the conduction in the atrioventricular canal retains its slow conducting properties, whereas the conduction velocity in the working myocardium increases (Figure 2) [10]. This gradually results in an adult-like electrocardiogram in which the initial base to apex activation will transform into apex to base activation with an atrioventricular delay, although at this stage a fibrous annulus fibrosis has not formed yet [11]. The left ventricle will be formed first from progenitor cells, followed by the right ventricle, and finally the definitive OFT. The progenitor cells of these three compartments have a different developmental history and have been exposed to different signals and gene programs prior to their differentiation [12]. The OFT myocardium is initially situated entirely to the right ventricle, whereas the atrioventricular canal is initially situated entirely above the left ventricle. The OFT myocardium will retain its primary myocardial phenotype and can be distinguished from the growing (ballooning) ventricles by its expression patterns of transcription factors [13]. It is likely that differences in the phenotype and epigenetic state of the mature right and left ventricles and OFT have their origin in these developmental processes. Mice and other small mammals are often used as models to study human cardiac diseases. It is therefore important to comprehend the similarities and differences in cardiac development and electrophysiology between mice and humans [13,14,15].

## 3. Development of the Pulmonary Vein Myocardium and Atrial Arrhythmias

### 3.1. Development of the Pulmonary Vein Myocardium

The pulmonary vein myocardium forms around E11.5 in mice (week 5 in human) from mesenchyme surrounding the dorsal atrial wall. This mesenchyme expresses the transcription factor Nkx2-5 and develops in close proximity to the caval vein myocardium that encompasses the developing sino-atrial node and does not express factor Nkx2-5 [16,17,18]. After initial differentiation, the newly formed myocardium proliferates and forms the myocardial sleeves surrounding the pulmonary veins. This proliferation step depends on the expression of the transcription factor Pitx2c [19]. Moreover, the myocardialization process may depend on the connection of the pulmonary veins to the left atrium. This is suggested by the absence of myocardial sleeves surrounding the pulmonary veins in patients with congenital heart disease in which the pulmonary veins fail to drain into the left atrium [20].

**Figure 3:**
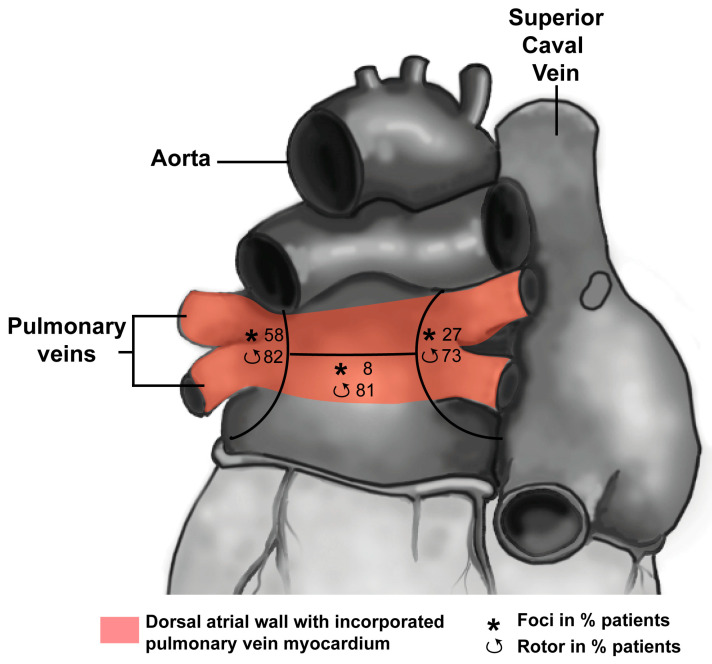
Distribution of drivers (focal breakthroughs, asterisk; reentry events, curved arrows) in three regions is reported as the percentage of patients (Redrawn from [40]). The pink area indicates the continuum of the left atrial dorsal wall and pulmonary veins after incorporation (for explanation see text). Note that ectopic foci are nearly absent in the roof of the left atrium.

In mice, as well as in humans around 8 weeks of development, the pulmonary veins drain in the common atrium through a solitary vessel. Lineage studies in mice suggest that pulmonary vein myocardium does not contribute to the formation of the atrial myocardium. In humans, however, this may be different as the walls of the initial solitary vessel will become incorporated into the left atrium around 15 weeks of development, resulting in up to four separate pulmonary venous orifices draining into the left atrium [21]. The total number of orifices may vary depending on the extent of incorporation. This suggests that the atrial myocardium in between the orifices of the pulmonary veins is composed of a mixture of vascular wall and myocardium that during development enclosed the pulmonary veins (pink area in Figure 3), providing it with a distinct developmental history compared to the remainder of the atria [22]. Nevertheless, the pulmonary vein and atrial myocardium both express the working myocardial gene program, indicated by absence of, e.g., Hcn4 and presence of Cx40 [23]. Action potential duration, however, is shorter and the upstroke velocity is lower in pulmonary vein compared to atrial myocytes [24]. The latter is not caused by reduced sodium current (I_Na_) but is the result of more depolarized resting membrane potential due to reduced expression of inward rectifier channels [25].

### 3.2. Pulmonary Vein Myocardium and Arrhythmias

Atrial fibrillation (AF) is the most prevalent arrhythmia in adults and is related to a high risk of stroke [26]. The pulmonary vein myocardium is often the origin of arrhythmia in AF patients [27]. Both structural and electrical remodeling are thought to play an important role in the development of AF [28,29]. In addition, several transcription factors determining pulmonary vein fate during development have been implicated in susceptibility to AF, and some reports suggest the presence of nodal-like cells in the myocardial sleeve of the pulmonary veins [30,31]. Mutations in NKX2-5 are found in a subset of patients with atrial fibrillation, together with several mutations in genes encoding for potassium channels [32,33]. In *Nkx2-5* haploinsufficient mice, expression of *Hcn4* is increased whereas that of *Gja5* (Cx40) is decreased in the pulmonary vein myocardium compared to their wild-type littermates. The ectopic activity seen in AF patients could be the result of the altered critical balance between phase 4 depolarization of the action potential (controlled by HCN4) and coupling between cells (modulated by Cx40) [27]. Similarly, PITX2C is an AF susceptibility locus [34]. The expression of PITX2C is decreased in patients with sustained AF when compared to healthy individuals, and mice haploinsufficient for *Pitx2c* show increased expression of ion channels linked to AF [35,36]. Furthermore, we have recently shown that Pitx2c modulates atrial electrophysiology through a T-box factor 5 (Tbx5)-dependent gene regulatory network involving *Scn5a*, *Gja1*, *Ryr2*, *Dsp*, and *Atp2a2* [37]. Abnormal calcium homeostasis also caused atrial arrhythmias in mice with reduced expression of Prrx1, a transcription factor that is related to AF in human [38,39]. Unravelling the role of loci (of transcription factors) provided by genome-wide association studies in altering the molecular make-up of atrial and pulmonary vein myocardium will contribute to the understanding of initiation and maintenance of AF and may foster future patient risk stratification.

## 4. Development of Atrioventricular Canal and Accessory Pathways Formation

### 4.1. Development of the Atrioventricular Canal

Slow conduction in the atrioventricular canal results from the absence of *Gja5* (Cx40) and *Gja1* (Cx43), which form gap junctions with high conductance, and the absence of *Scn5a* (Na_V_1.5, voltage gated sodium channel type V) [41]. In addition, *Gjd3* (Cx30.2 in mice; Cx31.9 in humans) is expressed in the atrioventricular canal and forms gap junctions with low conductance leading to slow conduction [41,42]. The expression of *Gjd3* (Cx30.2) in the atrioventricular canal is regulated by transcription factors Gata4 and Tbx5 [43]. The expression of *Gja5* (Cx40), *Gja1* (Cx43) and *Scn5a* in the atrioventricular canal is repressed by Tbx2 and Tbx3, which in turn are regulated by Wnt-, bone morphogenetic protein (Bmp) 2- and Notch signalling [9,44,45,46,47]. Not surprisingly, genome-wide association studies have linked genetic variations in (non)coding regions of these genes to QRS duration and PR interval in healthy individuals [48,49,50].

**Figure 4:**
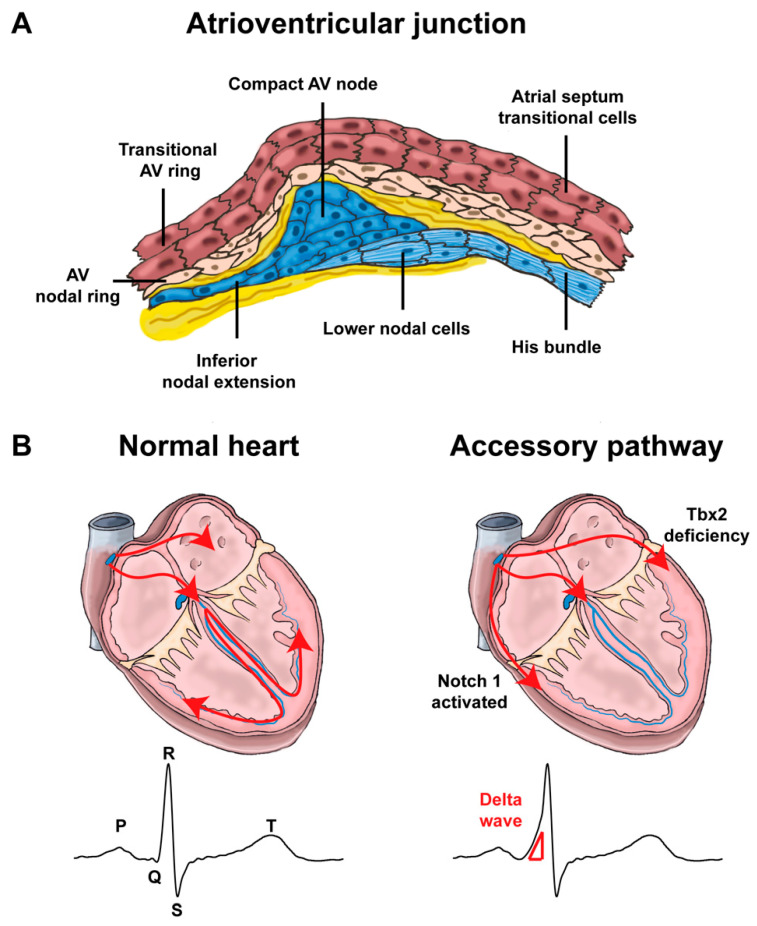
Atrioventricular junction and impulse propagation (**A**). Schematic representation of the different components of the atrioventricular junction (Inspired by [53,70]). Note that lower nodal cells share a common origin with the His bundle cells. (AV: atrioventricular) (**B**). In normal hearts, the electric impulses initiated by pacemaker cells in the sinoatrial node propagate through the atrial myocardium and trigger its contraction. At the atrioventricular node, the impulses are delayed for a period to facilitate alternating contraction of the atrial and ventricular myocardium. After the atrioventricular delay, the electrical impulses rapidly travel to the ventricular myocardium via the His-Purkinje system and stimulate the ventricular myocardium. In Notch1-activated and Tbx2-deficient hearts, accessory pathways are formed as a result of malformation of the atrioventricular canal myocardium, commonly right-sided in Notch1-activated mice and left-sided in Tbx2-deficient mice. Because of faster conduction through the accessory pathways than through the atrioventricular node, the ventricular myocardium is prematurely stimulated (preexcitation). The ECG shows a short PR interval, a slurred upstroke (“delta wave”) of the QRS complex and a widened QRS complex.

During early development, there is myocardial continuity between the atria and ventricles via the atrioventricular canal myocardium. Correct patterning of the atrioventricular canal is essential for initiating the formation of the annulus fibrosis, which will electrically insulate the atria and ventricles in the adult heart [41]. The annulus fibrosis will form from mesenchyme derived from the epicardium [51,52]. Most of the embryonic atrioventricular canal myocardium differentiates to ventricular working myocardium, whereas a small part forms the atrioventricular node and ring bundles [53,54,55]. Nkx2-5, Tbx5 and Notch-signaling are part of the transcriptional network underlying formation of the atrioventricular node [17,56,57,58]. The atrioventricular junction in the adult heart can be identified based on the expression of *Tbx3*, *Hcn4*, *Gjc1* (Cx45) and *Gjd3* (Cx30.2) in mice, and the absence of atrial natriuretic peptide (*Nppa*), *Gja5* (Cx40) and *Gja1* (Cx43) [9]. Working myocardial gene expression in the atrioventricular junction (e.g., *Scn5a*) tapers off towards the compact atrioventricular node, whereas the nodal genes (*Hcn4*, *Cacna1g*) show the complementary pattern (Figure 4A) [53,59]. This differential gene expression profile in the atrioventricular junction relates to the transitional cells, which have been found to be of crucial importance for impulse conduction [60]. It has been reported that *Gja1* (Cx43) and *Gja5* (Cx40) are expressed in the lower part of the compact atrioventricular node (lower nodal cells) [61]. However, based on their origin and gene expression pattern we rather consider these myocytes part of the atrioventricular bundle [53]. In mice, the atrial myocardium is directly connected to the compact atrioventricular node and part of the right atrioventricular ring bundle, which are often referred to as the inferior nodal extension and transitional zone, thereby creating the basis for what in humans is commonly referred to as the fast and slow atrioventricular nodal pathway [53]. Based on expression patterns of *Islet1*, cardiac *troponinI* and *Hcn4* in chick embryos, it has also been postulated that the myocardium of the sinus venosus contributes to nodal extensions or transitional cells of the atrioventricular node [62].

### 4.2. Mispatterning of the Atrioventricular Canal and Arrhythmias

#### 4.2.1. Atrioventricular Conduction Disorders

A high-degree of atrioventricular conduction block is life-threatening and is a major indication for pacemaker implantation [63]. Atrioventricular block may result from various causes, including increased collagen deposition within the compact atrioventricular node during ageing, a diseased His-Purkinje system or prominent vagal activity [15,64,65]. Mutations in *NKX2-5* and *TBX5* are also found in a subset of patients suffering from impaired atrioventricular conduction [33]. Moreover, genome-wide association studies have implicated several loci in atrioventricular conduction, including *TBX3/TBX5*, *NKX2-5*, *SOX5*, *WNT11*, *SCN10A*, *SCN5A*, *CAV1-CAV2*, and *MEIS1* [48,49,50,66]. Recent evidence points to an even finer regulation of transcription factors activity by regulatory elements in the DNA. In turn, transcription factors regulate in a dose-dependent manner the expression of their target genes [67,68]. For example, Tbx3 dosage controls downstream atrioventricular canal expression of calcium channel *CACNA1G*, thereby impacting on PR and QRS durations [69]. Thus, transcriptional regulators important for heart development play a major role in the function of the atrioventricular conduction system. Variations and mutations in these genes may impact on each component involved in PR interval (atria, atrioventricular node, atrioventricular bundle, branches and Purkinje fibers), but the difficulty lies in identifying which of these components has been affected.

#### 4.2.2. Re-Entry Tachycardias Involving the Atrioventricular Junction

Several types of arrhythmias involve atrioventricular conduction. In atrioventricular nodal re-entrant tachycardia (AVNRT), the slow and the fast pathway within the atrioventricular junction and part of the transitional myocytes in the atrium form a re-entrant circuit [60]. Which factors underlie the development of this type of arrhythmia is not known, although some familial cases of AVNRT suggest a heritable component [71,72]. Atrioventricular re-entrant tachycardia (AVRT) results from an accessory myocardial connection between the atrial and ventricular myocardium (Figure 4B). The myocardium of the accessory connection can have a slow conducting nodal (Mahaim) or a fast conducting working myocardial (Öhnell) phenotype [73]. An accessory connection can lead to ventricular preexcitation and AVRT, as seen in patients with the Wolff-Parkinson-White (WPW) syndrome [74,75]. In the presence of atrial fibrillation, preexcitation may cause ventricular fibrillation and sudden death.

The mechanism underlying the development of accessories pathways with a nodal phenotype is not fully understood. In the adult heart, the largest remnant of the embryonic atrioventricular canal is the dorsal-caudal portion of the right atrioventricular ring bundle. The right-caudal position and nodal properties of Mahaim bundles suggest that they are remnants or ill-localized atrioventricular canal tissue, or are caused by deficient remodeling of the RV inflow tract components during development [76]. Connections with a working myocardial phenotype may also be remnants of the atrioventricular canal myocardium and result from abnormal patterning of the embryonic atrioventricular canal [41,58]. Bmp2, Notch1 and Tbx2 (the latter being downstream of Bmp2) are important for correct patterning of the atrioventricular canal myocardium. Inactivation of Bmp-signaling or Tbx2, or activation of Notch-signaling in mice, causes ventricular pre-excitation (Figure 4B) [41,58,77]. Indeed, deletions have been found in *BMP2* and in *JAGGED1* (Notch ligand) in patients with WPW syndrome [3,4].

## 5. Developmental Basis for RVOT Arrhythmias

### 5.1. Development of the RVOT

**Figure 5:**
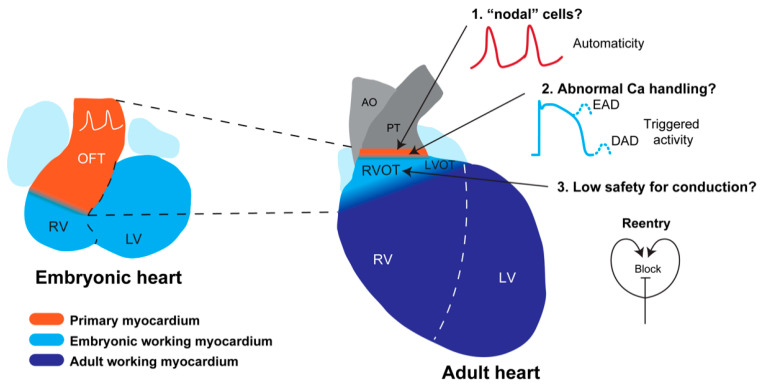
Developmental basis for RVOT arrhythmias. The adult RVOT has formed from the embryonic outflow tract (left), which is composed primary of myocardium exhibiting slow conduction and spontaneous activity. During development, the embryonic outflow tract acquires a working myocardial phenotype, e.g., fast conduction, and transforms into the RVOT. A small ring of primary myocardium, however, still remains just below the pulmonary valve, which may give rise to automaticity as seen in patients with idiopathic RVOT tachycardia. The myocardium of the free wall and septum of the adult RVOT has a working myocardial phenotype, although expression of Cx43 is lower than in the right ventricle. This may set the stage for re-entrant-based arrhythmias as seen in patients with the Brugada syndrome. Modified from [100] LV, left ventricle; RV, right ventricle; OFT, outflow tract; RVOT, right ventricular outflow tract; AO, aorta; PT, pulmonary trunk; LVOT, left ventricular outflow tract; Ca, calcium; EAD, early after depolarization; DAD, delayed after depolarization.

During the fetal period, the muscular part of the embryonic OFT will be incorporated into the right ventricular free wall and form the RVOT, whereas a small part will form the connection between the left ventricle and the aorta and form the LVOT (Figure 5) [78,79]. Thus, the RVOT and the LVOT have a common origin, which may point to a common mechanism underlying OFT arrhythmias. However, the inferior part of the embryonic OFT gives rise to the subpulmonary myocardium (corresponding to RVOT) and the superior part to the subaortic myocardium. These two parts show differential gene expression (e.g., inferior part expresses *Sema3C*), and the subpulmonary myocardium is specifically affected and possibly largely absent in *Tbx1* mutant mice [80,81]. Therefore, the RVOT and LVOT are not molecularly identical [82]. The RVOT and LVOT acquire the working myocardial phenotype just before birth [83]. The myocardium just below the valves, however, retains its primary phenotype similar to the atrioventricular ring myocardium around the entrance of the left and right ventricle [83].

### 5.2. Predisposition of the RVOT for Arrhythmias

Arrhythmias originate predominantly in the RVOT in idiopathic outflow tract tachycardia, Brugada syndrome. Arrhythmias in these cardiac pathologies usually do not occur at young age but rather in adulthood, indicating that postnatal development and maturation play an important role in disease development. The electrophysiological signature of the RVOT, however, develops prenatally and is different from that of the left and right ventricle [84]. The developmental history and phenotype of the RVOT are not intrinsically arrhythmogenic but may predispose to arrhythmias in the setting of an active pathological mechanism that progresses during life (Figure 5).

### 5.3. Brugada Syndrome

The Brugada syndrome is characterized by ST segment elevation in the right precordial leads of the electrocardiogram, highly fractionated local electrograms in the RVOT and ventricular arrhythmias [85,86,87,88]. The mechanism underlying these characteristics is debated, but evidence supporting conduction delay or block as a potential mechanism is accumulating. In 20–30% of the Brugada syndrome patients, a loss of function mutation in *SCN5A* has been found [86]. A reduction in I_Na_ itself, however, does not lead to the Brugada characteristics [89]. In contrast, reducing the I_Na_ is used to discriminate between patients who have the Brugada syndrome and patients who have not [86]. In patients with the Brugada syndrome, subtle small structural discontinuities have been demonstrated in the right ventricular wall and RVOT [90,91]. Experimental and clinical studies have shown that conduction can be delayed in myocardium with small structural discontinuities or even be blocked by a mechanism called current-to-load mismatch [92,93]. The conduction block is a prerequisite for re-entry and may generate a substrate for re-entrant based arrhythmias as seen in Brugada syndrome patients [90]. In addition, conduction delay or block can cause ST segment elevation on the body surface ECG, which is a hallmark of the Brugada syndrome [94,95].

Although a unifying mechanism explaining arrhythmias in Brugada syndrome patients has been proposed [95], it does not offer an explanation for the preferential location of these arrhythmias in the RVOT. We surmise that genes of the ventricular working myocardial gene program are less active in the RVOT, resulting in a reduced conduction reserve, thereby facilitating current-to-load mismatch and subsequently arrhythmias [84,96,97,98]. Indeed, lower expression of CX43 protein has been found in the epicardial region of the RVOT when compared to the right ventricle in patients with Brugada syndrome [99].

### 5.4. Idiopathic Outflow Tract Tachycardia

Idiopathic RVOT tachycardia are catecholamine-sensitive, suggesting automaticity or triggered activity as an underlying mechanism [101]. Accordingly, idiopathic arrhythmias can be treated with adenosine or beta-blockers [102]. A subset of myocytes in the RVOT have longer action potentials, do not repolarize fully to resting membrane potential, have a higher sarcoplasmic reticulum calcium content and easily develop early after depolarizations when compared to right ventricular myocytes [103,104]. This electrophysiological phenotype is expected from primary ring myocardium that is present just below, and above, the valves of the pulmonary artery. These primary myocytes may, in the presence of structural changes or uncoupling, give rise to spontaneous activity [105,106]. Consistently, ectopic beats in the RVOT are reported to originate from the myocardium just below the pulmonary valve and even from myocardial sleeves into the pulmonary artery [107,108]. For the moment, however, a direct relationship between these primary cells and idiopathic RVOT tachycardia remains hypothetical and further research is required to determine a causal relationship.

Typically, idiopathic RVOT tachycardia arises more frequently during periods of wakefulness, stress and activity while disappearing entirely during rest [101]. Current evidence strongly points towards a role for the autonomic nervous system, and in specific sympathovagal balance, to the initiation and maintenance of these arrhythmias. Studies in patients with idiopathic RVOT tachycardia showed that episodes of ventricular tachycardia were typically preceded by a sudden shortening of the RR interval on electrocardiogram, likely attributable to abrupt sympathetic predominance [109,110,111]. In canines, RVOT tachycardia induced by catheter-mediated high-frequency stimulation of the pulmonary artery originated most often from sites of the RVOT septal wall, the same sites that showed a greater density of tyrosine hydroxylase-positive (sympathetic) neurons compared to non-origin sites [112,113]. The RVOT septal wall also previously found the preferential site of occurrence of RVOT tachycardia in patients compared to other RVOT sites [114].

Other than its anatomical site of origin, the mechanism of idiopathic RVOT tachycardia can be considered well established and involves cAMP-mediated intracellular calcium overload that, in turn, predisposes it to afterdepolarizations and triggered activity [100,115,116]. In particular, genes involved in intracellular calcium regulation were differentially regulated in RVOT septal wall biopsies taken from patients with idiopathic RVOT tachycardia compared to control subjects, further indicating a calcium-dependent mechanism [117]. The distinctive electrophysiological properties of a subset of RVOT myocytes similar to that of the primary ring myocardium may predispose them to an increased triggered activity and tachycardia upon conditions of increased sympathetic tone.

## 6. Conclusions

In this review, we show a direct relation between the spatio-temporal activity of particular cardiac transcription factors and the electrophysiological characteristics of myocardial components. Insight into the mechanisms that regulate the expression of these factors and their consequences on the expression of their target ion channel genes could lead to the identification of new approaches and candidate markers for improved arrhythmogenic risk stratification.
